# Autophagy-Related Long Non-coding RNA Signature as Indicators for the Prognosis of Uveal Melanoma

**DOI:** 10.3389/fgene.2021.625583

**Published:** 2021-04-01

**Authors:** Yi Cui, Mi Zheng, Jing Chen, Nuo Xu

**Affiliations:** ^1^Department of Ophthalmology, Fujian Medical University Union Hospital, Union Clinical Medical College, Fujian Medical University, Fuzhou, China; ^2^Department of Ophthalmology, Fujian Provincial Hospital, Shengli Clinical Medical College, Fujian Medical University, Fuzhou, China

**Keywords:** long non-coding RNAs, autophagy, uveal melanoma, prognostic signature, The Cancer Genome Atlas, Gene Expression Omnibus

## Abstract

This study aimed to develop an autophagy-associated long non-coding RNA (lncRNA) signature to predict the prognostic outcomes of uveal melanoma (UM). The data of UM from The Cancer Genome Atlas (TCGA) were enrolled to obtain differentially expressed genes (DEGs) between metastasizing and non-metastasizing UM patients. A total of 13 differentially expressed autophagy genes were identified and validated in Gene Expression Omnibus, and 11 autophagy-related lncRNAs were found to be associated with overall survival. Through performing least absolute shrinkage and selection operator regression analyses, a six-autophagy-related lncRNA signature was built, and its efficacy was confirmed by receiver-operating characteristic, Kaplan–Meier analysis, and univariate and multivariate Cox regression analyses. A comprehensive nomogram was established and its clinical net benefit was validated by decision curve analysis. GSEA revealed that several biological processes and signaling pathways including Toll-like receptor signaling pathway, natural killer cell-mediated cytotoxicity, and B- and T-cell receptor signaling pathway were enriched in the high-risk group. CIBERSORT results showed that the signature was related to the immune response especially HLA expression. This signature could be deployed to assist clinicians to identify high-risk UM patients and help scientists to explore the molecular mechanism of autophagy-related lncRNAs in UM pathogenesis.

## Introduction

Uveal melanoma (UM) is the most common and aggressive malignant neoplasm of the eye in adults, and up to 50–62% of UM patients will suffer from metastasis (Kujala et al., 2003; Amaro et al., 2017; Kaliki and Shields, 2017). Local interventions including transpupillary thermotherapy (Mashayekhi et al., 2015), photodynamic thermotherapy (Cerman and Cekic, 2015), brachytherapy (Collaborative Ocular Melanoma Study Group, 2006), and surgical enucleation (Shields and Shields, 2015) are the main strategies to treat the primary tumor, but the overall prognosis for metastatic UM remains poor. Therefore, it is imperative to identify high-risk UM patients with poor prognosis. Clinical features including patient age, tumor apical height, involvement of ciliary body, and largest basal diameter (LBD) of the tumor have been identified as prognostic factors for metastasis (Chew et al., 2015; Correa and Augsburger, 2016). Recently, scientists have proposed certain molecular signatures as predictors of high-risk UM, such as the gene expression profile (GEP) model (Zhang et al., 2014), DNA methylation-based model (Li et al., 2018), microRNA (miRNA) model (Xin et al., 2019), and alternative splicing event model (Wan et al., 2020). However, the prognostic value of long non-coding RNAs (lncRNAs) in UM has not been fully explored.

LncRNAs are a set of RNAs with a transcript length of over 200 nucleotides and have limited or no protein-coding capacity. Previous data has shown that abnormal activation of lncRNAs participates in various biological processes and human tumors including UM. A number of studies have shown that aberrant lncRNA expressions like MALAT1, FTH1P3, ZNNT1, and CANT1 played important roles in the initiation and progression of UM (Xing et al., 2017; Zheng et al., 2017; Li et al., 2020; Wu et al., 2020). Recent evidence highlights the involvement of lncRNA ZNNT1 in the tumorigenesis of UM by regulating key autophagy gene expression (Li et al., 2020). Therefore, there exist some interactions between lncRNAs and autophagy processes that could contribute to the pathogenesis of UM.

Autophagy is a highly conserved intracellular degradation system required for the maintenance of homeostasis and promotion of survival when facing cellular stress. It plays critical roles in age-related diseases including cancers (Mizushima, 2007). According to previous studies, autophagy was proved to be upregulated in UM and to be associated with poor prognosis (Giatromanolaki et al., 2011). Specific autophagy inhibitors might serve as a potential approach to suppress UM (Zhang et al., 2016). On the other hand, increased autophagy could be protective and cooperate with apoptosis to induce cell death in UM (Ambrosini et al., 2013). Hence, exploring autophagy-related gene signatures may deepen the understanding of the underlying molecular mechanism of UM and provide insights into its prognosis.

Based on the above background, we postulated that autophagy-related lncRNAs might help us identify high-risk UM patients and customize their personalized targeted treatment. This study aimed to analyze the correlation between the differentiated expressed lncRNAs and autophagy-related genes in UM and to screen for the most survival-relevant autophagy-related lncRNA signature by least absolute shrinkage and selection operator (LASSO) regression. Furthermore, univariate and multivariate Cox regression analyses were used to confirm its prognostic value in UM. This study may provide new insights into prognostic markers and deepen the understanding of autophagy-related lncRNA in UM.

## Materials and Methods

### Data Resource and Preprocessing

**FIGURE 1:**
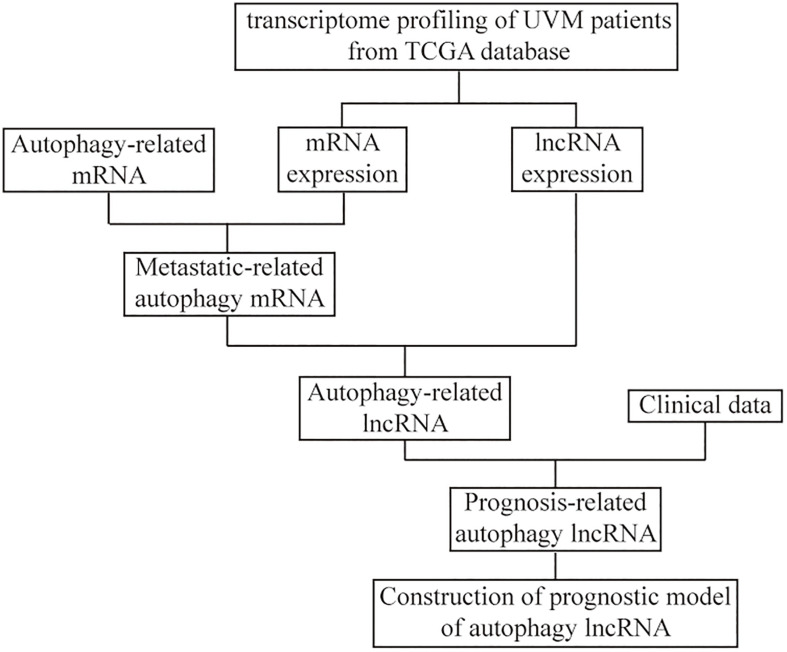
The flowchart of the construction of the prognostic model of autophagy long non-coding RNA (lncRNA) in uveal melanoma (UM).

The RNA expression profiles of 80 UM patients were downloaded in FPKM format from The Cancer Genome Atlas (TCGA) database using the GDC Data Transfer Tool^1^, and the mRNA and lncRNA expression data were also acquired. The relevant clinicopathological characteristics including sex, age, and cancer stage were downloaded from the UCSC Xena website^2^. The detailed clinical characteristics are shown in Table 1. The flowchart of the analysis procedure is shown in Figure 1. The gene expression data of GSE22138 that contains 63 tissue samples of UM patients were downloaded from the Gene Expression Omnibus (GEO) as a validation dataset (Laurent et al., 2011).

**TABLE 1:** Baseline data and correlations between risk score of lncRNA signature and clinicopathological characteristics in TCGA cohort.

Variables	All (*n* = 80)	Low risk (*n* = 40)	High risk (*n* = 40)	*P*-value
**Gender**				0.50
Female	35 (43.8%)	19 (47.5%)	16 (40.0%)	
Male	45 (56.2%)	21 (52.5%)	24 (60.0%)	
**Age**				0.03
<60	36 (45.0%)	23 (57.5%)	13 (32.5%)	
≥60	44 (55.0%)	17 (42.5%)	27 (67.5%)	
**Pathologic stage**				0.02
II	39 (48.8%)	15 (37.5%)	24 (60.0%)	
III	36 (45.0%)	20 (50.0%)	16 (40.0%)	
IV	4 (5.0%)	4 (10.0%)	0 (0.0%)	
**Type**				<0.001
Non-metastatic	61 (76.3%)	38 (95.0%)	23 (57.5%)	
Metastatic	19 (23.8%)	2 (5.0%)	17 (42.5%)	

### Identification of Differentially Expressed Genes and Autophagy-Related LncRNAs

In order to analyze the metastasis-related gene characteristics of UM patients, the differentially expressed genes (DEGs) between the metastasizing and non-metastasizing groups were calculated using the limma R package (Ritchie et al., 2015). DEGs with absolute log (fold change) > 0.05 and false discovery rate (FDR) < 0.05 were considered to be included for subsequent analysis. The result of differential analysis was presented in a heat map. Then, the autophagy-related gene set was downloaded from the MSigDB database^3^ (Subramanian et al., 2005), and the metastasis-related autophagy DEGs were extracted by the VennDiagram R package (Chen and Boutros, 2011). The expression correlations between those DEGs were calculated, and the protein–protein interaction (PPI) network was constructed by the Search Tool for the Retrieval of Interacting Genes (STRING) database^4^. The autophagy-related lncRNAs were identified based on the criteria that the correlation coefficient | *R*^2^| > 0.3 and *P* < 0.05.

### Construction of Prognostic Autophagy-Related LncRNA Signature

The LASSO method was used to further analyze the prognostic autophagy-related lncRNAs for dimensionality reduction and feature selection. The coefficients obtained from the LASSO regression were weighted by each normalized gene expression value to construct the prognostic signature model, and the risk score of each UM patient was calculated by the formula as follows:

$$\mathrm{RiskScore}=\sum_{i}\mathrm{Coefficient}\left({\text{hubgene}}_{i}\right)*\mathrm{lncRNA}\mathrm{Expression}\left({\text{hubgene}}_{i}\right)$$

Based on the median risk score, UM patients in the TCGA cohort were divided into high-risk and low-risk subgroups. Then, univariate and multivariate Cox regression analyses were performed to analyze the predictive ability of the signature model and patients’ clinical–pathological parameters on overall survival (OS) and progression-free interval (PFI). A prognostic nomogram integrating risk scores and clinical–pathological characteristics was constructed to analyze the probable 1-, 3-, and 5-year OS of the UM patients. Harrell’s concordance index (C-index) was applied to evaluate the predictive accuracy of the nomogram. To determine the clinical predictive validity of the nomogram, decision curve analysis (DCA) was used to quantify the net benefits at threshold probabilities in the dataset.

### Functional Enrichment Analysis and Gene Set Enrichment Analysis

Gene Ontology (GO) and the Kyoto Encyclopedia of Genes and Genomes (KEGG) pathway enrichment analyses were performed by ClusterProfiler R package (Yu et al., 2012). FDR < 0.05 was considered statistically significant. To investigate the differences in biological processes between the metastasizing and non-metastasizing groups, gene set enrichment analysis (GSEA) was performed between the two subgroups. The “c2.cp.kegg.v6.2.symbols.gmt” gene set was chosen as the reference gene list (Subramanian et al., 2005). We performed 1,000 times of permutations each time, and a pathway was considered to be significantly upregulated with FDR < 0.1. Furthermore, we analyzed the correlation between different subgroups and some related biologically processes constructed by Mariathasan et al. (2018), which included (1) immune checkpoints; (2) antigen processing processes; (3) CD8+ T-effector features; (4) epithelial–mesenchymal transition (EMT) markers, including EMT1, EMT2, and EMT3; (5) angiogenesis features; (6) pan-fibroblast TGF-β response signature (Pan-FTBRS); (7) WNT targets; (8) DNA damage repair; (9) mismatch repair; (10) nucleotide excision repair; (11) DNA replication; and (12) antigen processing and presentation.

### Comparison of Immune Cell Infiltration Levels and Immune Scores Between the High-Risk and Low-Risk Subgroups of UM Patients

To quantify the proportion of different immune cells in the UM samples, the CIBERSORT algorithm and the LM22 gene set were used to analyze 22 human immune cell phenotypes in the tumor immune microenvironment (TME) (Newman et al., 2015). CIBERSORT is an algorithm for calculating the cell-type proportions in tumor samples, which uses a set of reference gene expression values as a representation (consisting of 547 marker genes). The Mann–Whitney *U* test was used to compare the infiltration level of immune cells between the two groups.

ESTIMATE (Estimation of STromal and Immune cells in MAlignant Tumor tissues using Expression data) is an algorithm for quantifying the immune infiltration level in a tumor sample based on gene expression data (Yoshihara et al., 2013). We calculated the immune and stromal scores of each UM sample using the ESTIMATE R package and compared the expression differences of human leukocyte antigen (HLA) family genes between the two groups.

### Survival Analysis

The effects of the signature model on OS and PFI were compared. The Kaplan–Meier survival curve by survival package was used to perform survival analysis, and the log-rank test was used to assess the significance of the difference in survival time between the two groups.

### Statistical Analysis

All data processing and analysis were performed using the R software (version 3.6.2). For the comparison of the two sets of continuous variables, the statistical significance of normally distributed variables was estimated by independent Student’s *t*-test, and differences between non-normally distributed variables were analyzed by Mann–Whitney *U* test. Categorical variables in the two groups were compared and analyzed using the chi-squared test or Fisher’s exact test. Pearson correlation analysis was used to calculate correlation coefficients between different genes. Univariate and multivariate Cox regression analyses were performed to identify independent prognostic factors. The pROC R package was used to visualize receiver-operating characteristic (ROC) curves, and the area under the curve (AUC) was calculated to assess the prognostic accuracy of the risk score (Robin et al., 2011). *P* < 0.05 was considered to be statistically significant.

## Results

### Identification of Metastasis-Associated Autophagy DEGs

**FIGURE 2:**
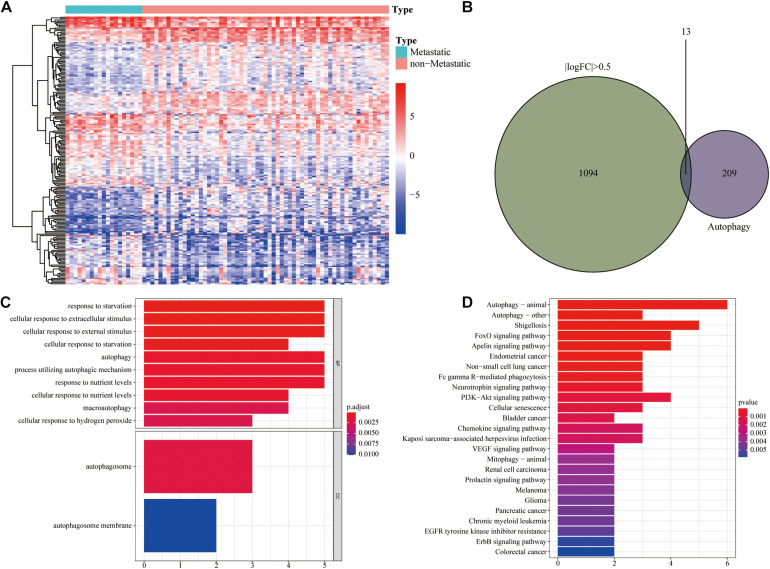
Identification of autophagy differentially expressed genes (DEGs). **(A)** Heat map of the 1,107 DEGs between metastatic and non-metastatic UM. **(B)** Venn diagram of 13 autophagy DEGs. **(C)** GO functional enrichment analysis of autophagy DEGs. **(D)** KEGG pathway analysis of autophagy DEGs.

To obtain metastasis-associated signature genes in UM patients, the gene expression profiles of metastasizing and non-metastasizing UM patients were analyzed and 1,107 DEGs were identified [| log (fold change)| > 0.5 and FDR < 0.05 filters, Figure 2A]. Meanwhile, the Venn diagram showed that there were 13 autophagy DEGs (Figure 2B). GO and KEGG analyses of all DEGs are shown in Supplementary Figure 1. GO and KEGG analyses of autophagy DEGs revealed that these DEGs were mainly enriched in cellular responses to starvation and extracellular stimulus and in relevant autophagy processes (Figures 2C,D).

**FIGURE 3:**
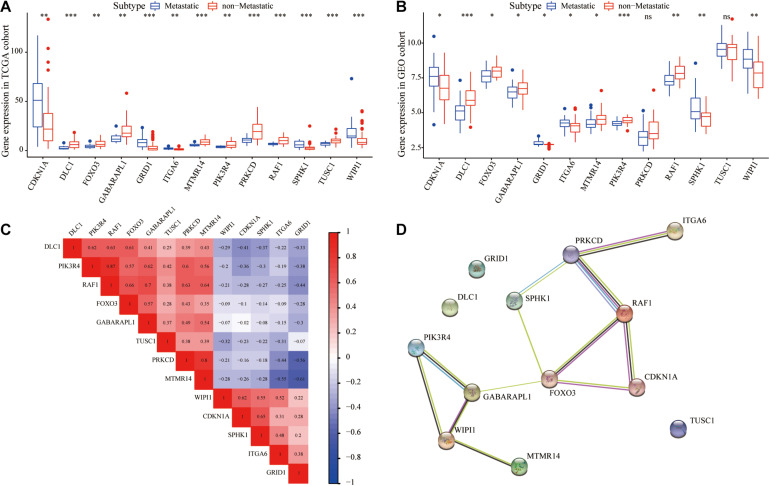
Validation and correlation analysis of autophagy DEGs. **(A)** Box plot of the expression of the autophagy DEGs between metastatic and non-metastatic UM in TCGA cohort. **(B)** Box plot of the expression of the autophagy DEGs between metastatic and non-metastatic UM in the GEO cohort. **(C)** Correlation analysis diagram of autophagy DEGs. **(D)** Protein–protein interaction network of autophagy DEGs. *P < 0.05, **P < 0.01, ***P < 0.001.

We further analyzed the interrelationship between metastasis-associated autophagy DEGs. As shown in Figures 3A,B, all 13 autophagy DEGs were differentially expressed with good concordance both in the TCGA and GEO cohorts. The correlation analysis showed significant correlations between the expression of most of these genes, with the highest correlation coefficient between RAF1 and PIK3R4 (Figure 3C). Meanwhile, the PPI network further revealed the interactions between 13 autophagy DEGs (Figure 3D).

### Identification of Prognostic Autophagy-Related LncRNAs in UM

**FIGURE 4:**
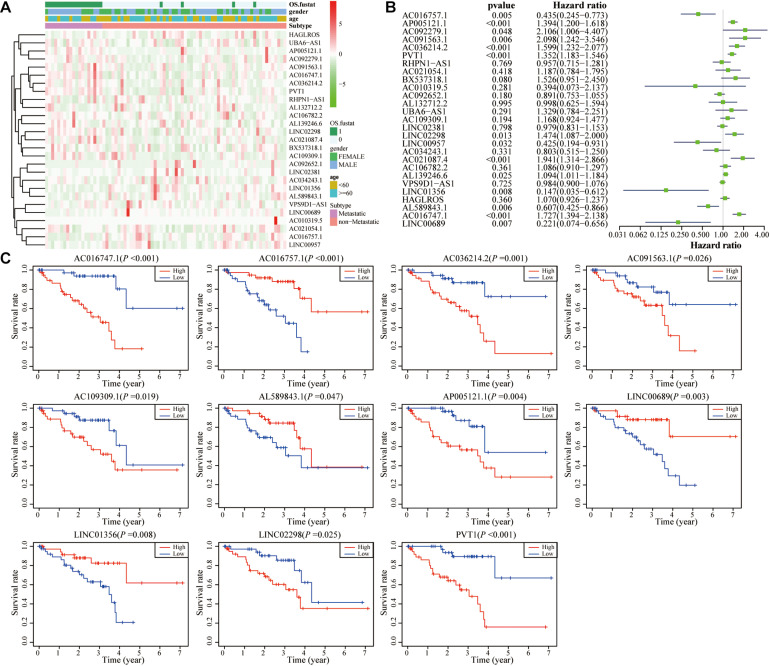
Identification of autophagy-related lncRNAs in UM. **(A)** Heat map of the autophagy-related lncRNAs and their correlation with clinical characteristics. **(B)** Univariate Cox regression analysis results show that 14 autophagy-related lncRNAs correlate with overall survival of UM patients. **(C)** Kaplan–Meier survival curve analysis shows that 11 autophagy-related lncRNAs correlated with overall survival of UM patients.

As shown in Figure 4A and Supplementary Figure 2, 27 autophagy-related lncRNAs were identified. The univariate Cox regression analysis showed that 14 lncRNAs had statistical significance, among which five lncRNAs were considered as protective factors and nine lncRNAs were considered as risk factors (Figure 4B). In order to confirm the impacts of these lncRNAs on the UM prognosis, all UM patients were ranked according to each lncRNA expression and subsequently divided into low and high groups (each group *n* = 40) according to media value. The survival prognostic analysis showed that 11 lncRNAs had statistically significant impacts on the prognosis of UM patient (Figure 4C).

### Construction of Prognostic Autophagy-Related LncRNA Signature

**FIGURE 5:**
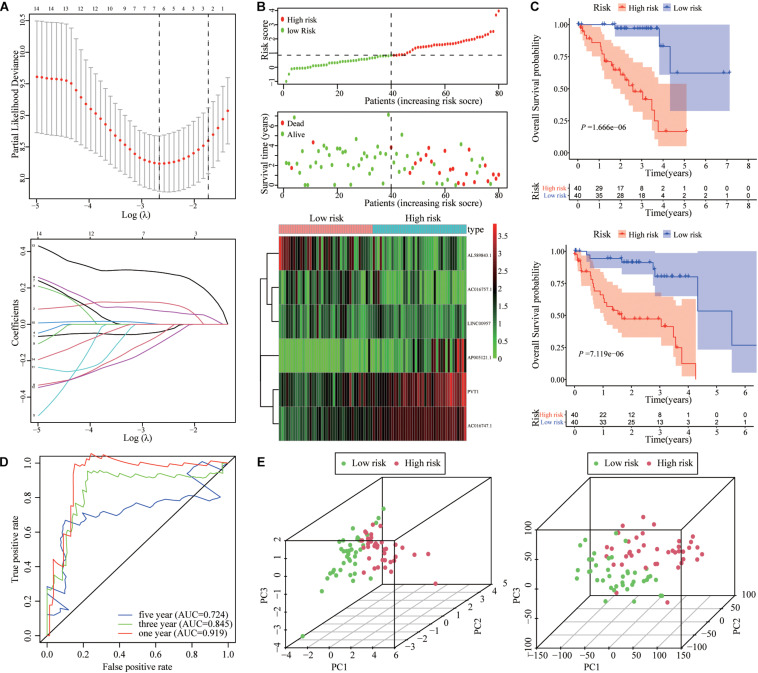
Establishment and validation of autophagy-related lncRNA signature. **(A)** LASSO coefficient profiles of lncRNA signature for overall survival (OS) in TCGA cohort. **(B)** Risk score distribution, survival status scatter plots, and heat map of the six prognostic lncRNAs in the high-risk and low-risk groups. **(C)** Kaplan–Meier analysis showed that UM patients in the high-risk group had poorer OS and PFI compared with UM patients in the low-risk group. **(D)** Time-dependent ROC curve of the lncRNA signature at 1, 3, and 5 years. **(E)** PCA of the lncRNA signature in high-risk and low-risk groups.

To quantify the impact of autophagy-related lncRNAs on the prognosis of each UM patient, we constructed a prognostic signature by LASSO regression analysis. As shown in Figure 5A, six hub autophagy-related lncRNAs were included in the prognostic signature model. The risk score for each UM patient was calculated using the coefficients of each gene obtained from the LASSO algorithm. The distribution and scatter plot of risk scores, survival curves, and gene expression plots for each patient are shown in Figure 5B.

Kaplan–Meier analysis showed that the OS and PFI of patients with high-risk scores were significantly shorter than those with low-risk scores (Figure 5C). The ROC results validated the prognostic accuracy of the signature model (Figure 5D). The area under the ROC curves (AUCs) of 1, 3, and 5 years were 0.919, 0.845, and 0.724, which confirmed the accuracy of the signature in predicting survival times of UM patients. The principal component analysis (PCA) showed that the signature model could separate the distribution patterns between high- and low-risk groups in terms of the six autophagy-related lncRNAs and all genes (Figure 5E).

### Clinical Correlation Analysis of the Signature and Nomogram Building

**FIGURE 6:**
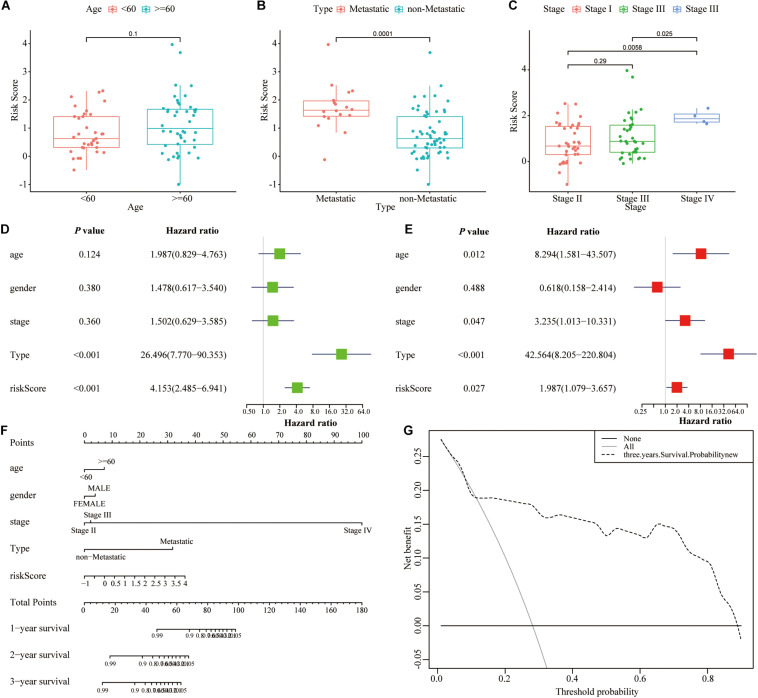
Nomogram for predicting the overall survival probability of patients with UM. **(A–C)** The relationships between risk score of lncRNA signature and clinical parameters including age, metastasis, and stage. **(D,E)** Univariate and multivariate Cox regression analysis for risk score of lncRNA signature and other clinicopathological characteristics. **(F)** The nomogram was established based on lncRNA signature and other clinicopathological characteristics for predicting OS in UM patients at 1, 3, and 5 years. **(G)** DCA curves of the nomogram in UM patients at 3 years.

The clinical correlation analysis showed that high-risk patients demonstrated a higher risk of metastasis and poorer stage compared with low-risk patients, whereas no significant correlation with patient’s age was found (Figures 6A–C). The univariate and multivariate Cox regression analyses showed that the lncRNA signature was an independent prognostic indicator for UM patients (Table 2 and Figures 6D,E). Then we constructed a nomogram that integrates clinicopathological characteristics and the signature to predict OS of UM patient (Figure 6F). C-index was used to calculate the discriminative power of the nomogram, which showed a high degree of discrimination [0.93 (0.975–0.885)]. The DCA showed a prognostic benefit in 18–95% of patients using the signature model (Figure 6G).

**TABLE 2:** Univariate and multivariate Cox analysis of prognostic lncRNA signature and clinical features for OS in TCGA cohort.

Variables	Univariate Cox analysis		Multivariate Cox analysis	
	HR (95% CI)	*P*-value	HR (95% CI)	*P*-value
Age (≥60 vs. <60)	1.99(0.83–4.76)	0.124	8.29(1.58–43.51)	0.012
Gender (male vs. female)	1.48(0.62–3.54)	0.380	0.62(0.16–2.41)	0.488
Stage (III + IV vs. I + II)	1.50(0.63–3.58)	0.360	3.24(1.01–10.33)	0.047
Type (metastatic vs. non-metastatic)	26.49(7.70–90.35)	<0.001	42.56(8.20–220.80)	<0.001
RiskScore (high vs. low)	4.15(2.49–6.94)	<0.001	1.99(1.08–3.66)	0.027

### Gene Set Enrichment Analysis and Immune Infiltration Analysis of the Signature Model

**FIGURE 7:**
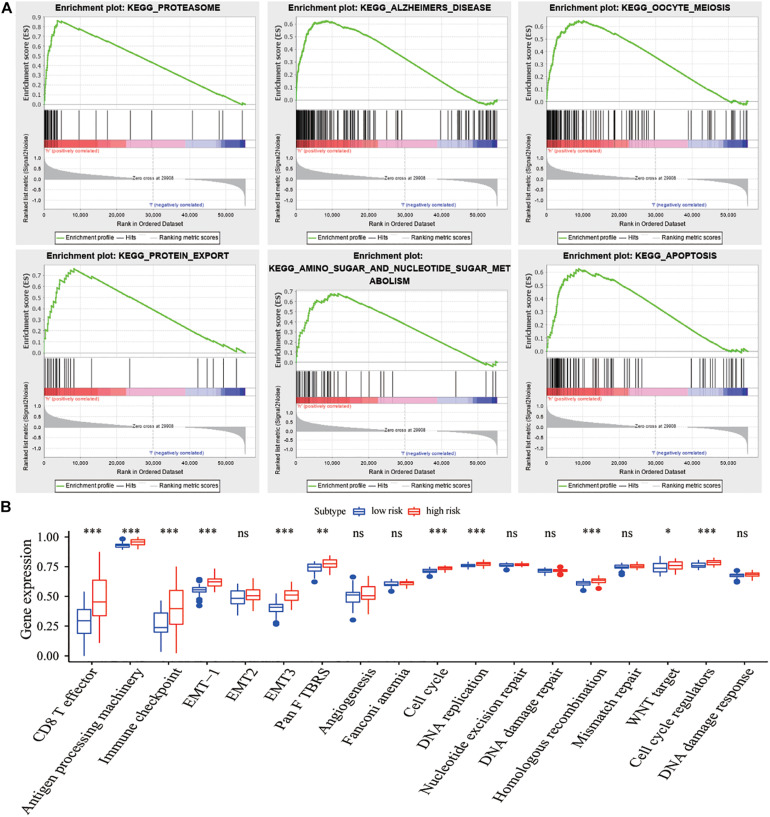
Functional enrichment analysis of lncRNA signature. **(A)** Six representative KEGG pathways in the high-risk group *via* GSEA. **(B)** Comparisons of different pathway features between the high-risk and low-risk groups. *P < 0.05, **P < 0.01, ***P < 0.001.

GSEA results showed that the proteasome, protein transport, metabolism, and apoptosis pathways were significantly enriched in the high-risk group compared with those in the low-risk group (Table 3 and Figure 7A). Meanwhile, some biologically relevant pathways, such as EMT, DNA replication, and cell cycle regulatory factors (Figure 7B), were significantly different between the high- and low-risk groups (*P* < 0.05).

**TABLE 3:** Results of the GSEA base on prognostic lncRNA signature.

Name	Size	Enrichment score	NES	FDR	Leading edge
KEGG_PROTEASOME	46	0.86	1.89	0.010	Tags = 78%, list = 7%, signal = 84%
KEGG_ALZHEIMERS_DISEASE	165	0.63	1.79	0.036	Tags = 54%, list = 15%, signal = 63%
KEGG_OOCYTE_MEIOSIS	112	0.65	1.77	0.030	Tags = 54%, list = 16%, signal = 65%
KEGG_PROTEIN_EXPORT	24	0.76	1.72	0.061	Tags = 71%, list = 15%, signal = 83%
KEGG_AMINO_SUGAR_AND_NUCLEOTIDE_SUGAR_METABOLISM	43	0.68	170	0.069	Tags = 77%, list = 21%, signal = 97%
KEGG_APOPTOSIS	87	0.63	1.69	0.073	Tags = 54%, list = 16%, signal = 64%
KEGG_PYRIMIDINE_METABOLISM	98	0.57	1.68	0.067	Tags = 50%, list = 17%, signal = 60%
KEGG_VIBRIO_CHOLERAE_INFECTION	54	0.63	1.68	0.061	Tags = 50%, list = 18%, signal = 61%
KEGG_GLYCOSAMINOGLYCAN_BIOSYNTHESIS_CHONDROITIN_SULFATE	22	0.70	1.68	0.054	Tags = 55%, list = 18%, signal = 66%
KEGG_CELL_CYCLE	124	0.64	1.68	0.049	Tags = 65%, list = 18%, signal = 79%

**FIGURE 8:**
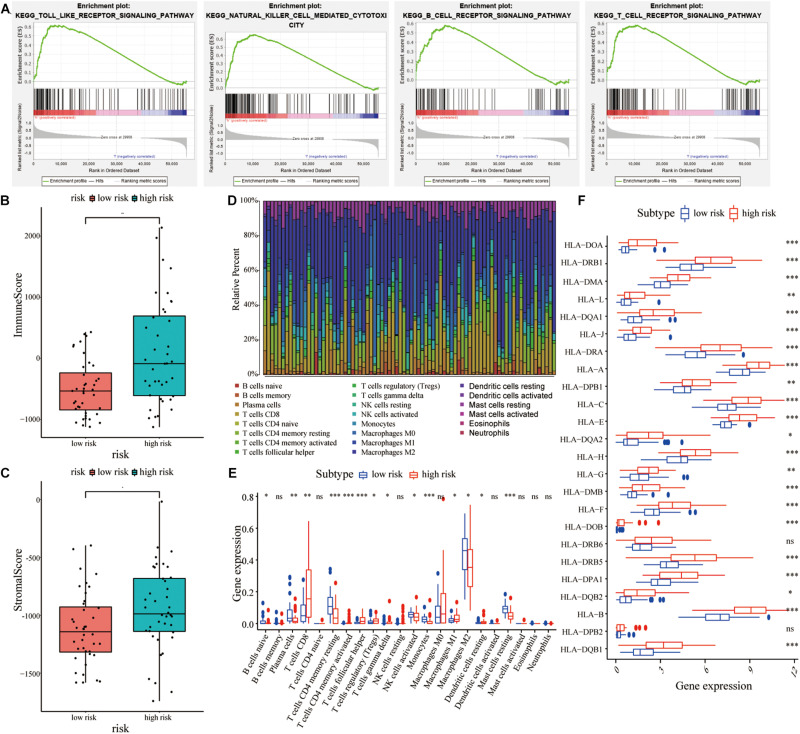
Correlations between lncRNA signature and immune cell infiltration in UM patients. **(A)** GSEA showed that various immune-related signaling pathways were enriched in the high-risk group compared with the low-risk group. **(B,C)** Comparisons of immune score and stromal score between the high-risk and low-risk groups. **(D)** Histogram of the 22 different specific immune cell infiltration in each UM samples. **(E)** Comparisons of various cell types in immune infiltration between the high-risk and the low-risk groups. **(F)** Comparisons of various HLA family genes between the high-risk and low-risk groups. *P < 0.05, **P < 0.01, ***P < 0.001.

GSEA results also showed that the change in DEGs was significantly enriched in immune-related signaling pathways, including Toll-like signaling pathway, natural killer cell signaling pathway, etc (Figure 8A). Therefore, we further analyzed the immune infiltration between the high- and low-risk groups. As shown in Figures 8B,C, the high-risk patients had significantly higher immune scores and stromal scores compared with the low-risk patients. To further analyze the infiltration of immune cells in UM patients, the gene expression data of UM patients were downloaded from the TCGA cohort and 22 different specific immune cell infiltration in each patient were obtained using the CIBERSORT algorithm (Figure 8D). As shown in Figure 8E, most of the immune cells were significantly different between the two groups (Figure 8E). Furthermore, the gene expression of the HLA family was significantly different between the two groups (Figure 8F).

## Discussion

The mortality rate of UM patients with advanced stage is higher than 95% within 5 years, and the survival of patients with metastasis UM is below 1 year (Kujala et al., 2003), so finding novel and effective prognostic biomarkers is critical for UM. Although some epigenetic signatures like miRNA signature and DNA methylation signature have been established to precisely identify high-risk UM, the lncRNA signature for UM has not been reported. Experimental studies have shown that lncRNAs play a critical role in UM by regulating many cellular processes including autophagy. Therefore, it may be helpful to build an autophagy-related lncRNA signature to predict the prognosis of UM and improve therapeutic approaches.

In the present study, two datasets (TGGA and GSE22138) were enrolled to construct novel prognostic autophagy-related lncRNA signatures for UM. After comparing DEGs between metastasizing and non-metastasizing UM patients, we screened out 13 autophagy genes and further identified 27 autophagy-related lncRNAs through establishing the lncRNA–autophagy gene co-expression network. Moreover, we utilized the LASSO regression mode to build a six-hub autophagy-related lncRNA model that could differentiate high- and low-risk UM patients. The survival analyses, ROC curve, and PCA analyses confirmed the accuracy of this model. Through univariate and multivariate Cox regression analyses, we also revealed that the risk score of this model was an independent factor in predicting prognosis of UM patients. This six-lncRNA model may guide in the selection of more precise and individualized treatment strategies for UM patients.

Through bioinformatics analysis, we identified 13 metastasis-related autophagy DEGs, namely CDKN1A, DLC1, FOXO3, GABARAPL1, GRID1, ITGA6, MTMR14, PIK3R4, PRKCD, RAF1, SPHK1, TUSC1, and WIPI1. Among them, FOXO3, which acts as a transcription factor to correct autophagy perturbations, was reported to attenuate proliferation, invasion, and migration and promote cellular apoptosis of UM (Yan et al., 2016, 2018). PRKCD was shown to suppress autophagy and implicated as a link to activate MAPK in GNAQ mutant UM (Chen et al., 2017; Zhang et al., 2017). Meanwhile, we found that RAF1 and PIK3R4 showed the highest correlation coefficient. RAF1 acts as a regulatory link between the membrane-associated Ras GTPases and the MAPK/ERK pathway, and this critical regulatory link can lead to inhibition of cell proliferation and induction of apoptosis in UM cells (Samadi et al., 2012). PIK3R4 belongs to the regulatory subunit of the PI3K complex that is involved in the maturation of autophagosomes and endocytosis. A previous study revealed that somatic mutation of PIK3R4 contributed to melanoma metastasis (Shull et al., 2012), and its functions in UM progress are worthy of further study. It is worth mentioning that only 13 DEGs out of 1,107 genes in our results were found to be autophagy-related genes, and only 11 autophagy-related lncRNAs were associated with overall survival for UM. However, the autophagy-related lncRNA signature we built was proven to be highly effective for predicting the overall survival for UM. These results may be explained by the fact that autophagy takes part in a certain but crucial phase of the metastatic cascade, but other complicated mechanisms take up more roles in regulating UM metastasis.

Autophagy is a lysosomal catabolic process that is regulated by non-coding RNAs such as miRNAs and lncRNAs. Experimental and clinical studies revealed that autophagy-related lncRNAs had significant diagnostic and therapeutic values in various cancers including UM. For example, a recent research reported that lncRNA ZNNT1 could act as a tumor inhibitor in UM by upregulating ATG12 gene expression, indicating delicate interactions between lncRNAs and autophagy (Li et al., 2020). Our model enrolled six autophagy-related lncRNAs including PVT1 that predicted overall survival in UM. PVT1 has been identified as a prognostic biomarker and played a critical role in the development of pancreatic ductal adenocarcinoma *via* triggering cytoprotective autophagy (Huang et al., 2018), and it affected EMT and cell proliferation and migration *via* modulating CDKN1A expression in breast cancer (Wang et al., 2018). Furthermore, PVT1 also took part in drug resistance of pancreatic cancer by interacting with ATG14 and promoting PtdIns3K activity (Zhou et al., 2020). In UM, silencing PVT1 could suppress tumorigenesis by activating the miR-17-3p-dependent p53 signaling pathway (Wu et al., 2019). Further in-depth studies are needed to investigate the molecular mechanism between PVT1 and autophagy in UM.

In order to explore the biological functions of the prognostic six-lncRNA model in UM, GSEA analysis was performed to compare the low- and high-risk groups. The results demonstrated that these lncRNAs might exert influence on UM progression through the immune-related pathway. LncRNAs are involved in immunological processes in cancers by mediating the functional state of immune cells and related pathways. Based on this lncRNA model, we found that the immune scores and stromal scores in high-risk UM were higher than in low-risk UM, and various members of the HLA family showed statistical difference between the two groups of UM. Several studies have shown an abnormal expression of the HLA family in UM and confirmed that a high expression of HLA class I is a risk factor for the development of metastasis (Gezgin et al., 2017; Souri et al., 2019; Wierenga et al., 2019). Recent studies showed that the lncRNA MIR155 host gene (MIR155HG) can be a potential prognostic marker for predicting the curative effect of immune checkpoint inhibitor therapy in multiple cancers including UM (Peng et al., 2019). Our results, combined with these publications, indicated that the six autophagy-related lncRNAs might play crucial roles in the pathogenesis of UM by regulating related genes to affect the immune responses, and there existed close relationships between autophagy and immune regulation in UM.

This study was not without limitations. All results of this study are based on bioinformatics analysis, and the prognostic signature enrolled some autophagy-related lncRNAs that no research confirmed their roles in UM so far. Thus, further experiments combining biochemical experiments and clinical prognostic data are required to confirm how these lncRNAs affect the prognosis of UM patients through autophagy exactly.

## Conclusion

In conclusion, this study screened and identified 13 dysregulated autophagy-related genes in UM, and a prognostic risk signature enrolling the six dysregulated autophagy-related lncRNAs was constructed, which was proven to be a useful marker for predicting UM patients’ prognosis with high efficacy. Based on the prognostic lncRNA model, dysregulated immune-related pathway, especially HLA expression, may contribute to the poor prognosis in high-risk UM. This signature model could be deployed to assist clinicians to identify high-risk UM patients and help scientists to explore the molecular mechanism of autophagy-related lncRNA in UM pathogenesis.

## Data Availability Statement

All datasets generated for this study are included in the article/Supplementary Material, further inquiries can be directed to the corresponding author/s.

## Author Contributions

YC and NX: conceptualization. YC: methodology and writing—original draft preparation. YC and MZ: formal analysis and investigation. NX and JC: writing—review and editing. NX, JC, and YC: funding acquisition. NX: supervision. All authors contributed to the article and approved the submitted version.

## Conflict of Interest

The authors declare that the research was conducted in the absence of any commercial or financial relationships that could be construed as a potential conflict of interest.

## Footnotes

1 https://portal.gdc.cancer.gov/

2 http://xena.ucsc.edu/

3 https://www.gsea-msigdb.org/gsea/msigdb

4 https://www.string-db.org/

## Supplementary Material

Supplementary Figure 1 GO and KEGG analyses of all DEGs. **(A)** GO functional enrichment analysis of all DEGs. **(B)** KEGG pathways analysis of all DEGs.

Supplementary Figure 2 Correlation analysis diagram between autophagy-related genes and lncRNAs.
