# Supplementary figures and images for: Autophagy-Related Long Non-coding RNA Signature as Indicators for the Prognosis of Uveal Melanoma
Source: Front Genet. 2021 Apr 1;12:625583. doi: 10.3389/fgene.2021.625583 (PMC8047156; doi:10.3389/fgene.2021.625583)

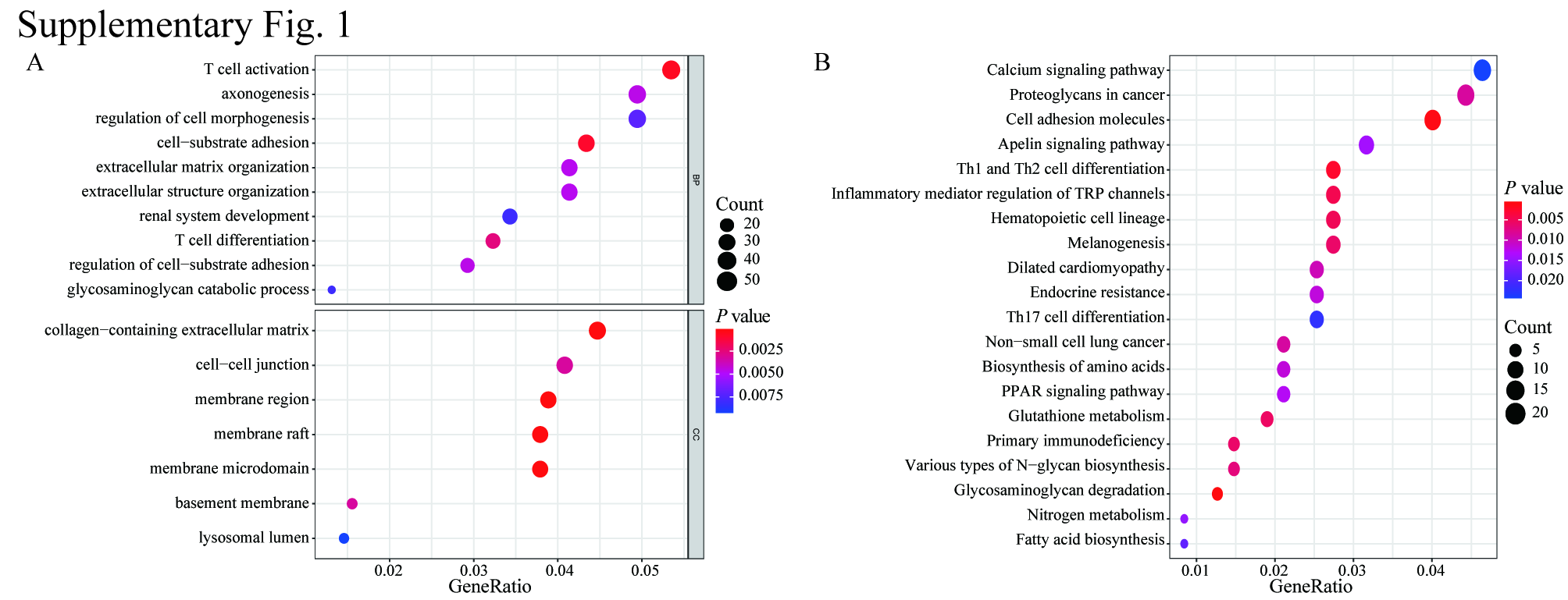

Supplement: Supplementary Figure 1 — GO and KEGG analyses of all DEGs. (A) GO functional enrichment analysis of all DEGs. (B) KEGG pathways analysis of all DEGs. [file Image_1.TIF]

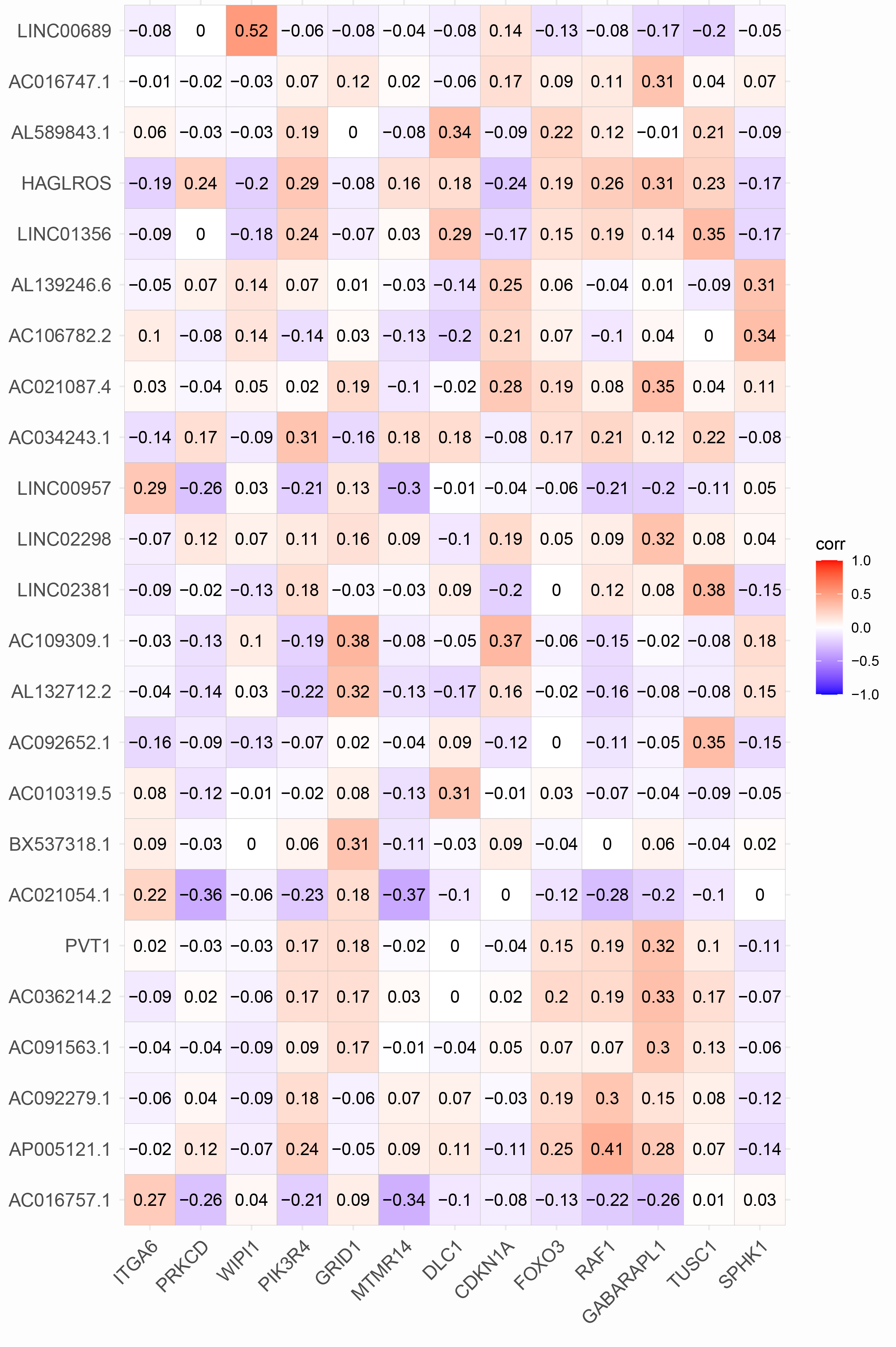

Supplement: Supplementary Figure 2 — Correlation analysis diagram between autophagy-related genes and lncRNAs. [file Image_2.TIF]
